# Sympatrically breeding congeneric seabirds (*Stercorarius* spp.) from Arctic Canada migrate to four oceans

**DOI:** 10.1002/ece3.8451

**Published:** 2021-12-21

**Authors:** Autumn‐Lynn Harrison, Paul F. Woodard, Mark L. Mallory, Jennie Rausch

**Affiliations:** ^1^ Migratory Bird Center Smithsonian Conservation Biology Institute, National Zoological Park Washington District of Columbia USA; ^2^ Canadian Wildlife Service, Northern Region Yellowknife NT Canada; ^3^ Department of Biology Acadia University Wolfville NS Canada

**Keywords:** Arctic, animal tracking, migration, nomadism, seabirds

## Abstract

Polar systems of avian migration remain unpredictable. For seabirds nesting in the Nearctic, it is often difficult to predict which of the world's oceans birds will migrate to after breeding. Here, we report on three related seabird species that migrated across four oceans following sympatric breeding at a central Canadian high Arctic nesting location. Using telemetry, we tracked pomarine jaeger (*Stercorarius pomarinus*, *n *= 1) across the Arctic Ocean to the western Pacific Ocean; parasitic jaeger (*S*. *parasiticus*, *n *= 4) to the western Atlantic Ocean, and long‐tailed jaeger (*S*. *longicaudus*, *n *= 2) to the eastern Atlantic Ocean and western Indian Ocean. We also report on extensive nomadic movements over ocean during the postbreeding period (19,002 km) and over land and ocean during the prebreeding period (5578 km) by pomarine jaeger, an irruptive species whose full migrations and nomadic behavior have been a mystery. While the small sample sizes in our study limit the ability to make generalizable inferences, our results provide a key input to the knowledge of jaeger migrations. Understanding the routes and migratory divides of birds nesting in the Arctic region has implications for understanding both the glacial refugia of the past and the Anthropocene‐driven changes in the future.

## Graphical abstract

For seabirds nesting in the Nearctic, it is often difficult to predict which of the world's oceans birds will migrate to after breeding. Here, we report on three related predatory seabird species (*Stercorarius* spp.) that divided across four oceans following sympatric breeding at a central Canadian high Arctic nesting location. We also report on extensive nomadic movements over ocean during the postbreeding period (19,002 km) and over land during the prebreeding period (5578 km) by pomarine jaeger, an irruptive species whose full migrations have been a mystery.

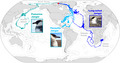

## 1 INTRODUCTION

Radar and biologging technology (Alerstam et al., 2007; Egevang et al., 2010) have provided new knowledge about polar systems of avian migration. But in the Nearctic, routes and destinations remain unpredictable. For seabirds, it is often difficult to predict which of the world's oceans birds will migrate across or to from their Arctic nest sites (Davis et al., 2016; Gutowsky et al., 2020; Mehl et al., 2004). Migratory patterns are the legacy of millions of years of changing lands and seascapes, glaciation events, intra‐ and interspecific competition, and speciation (Johnson & Herter, 1990). Studying the migratory connectivity of related species—the linking of migratory individuals or populations between different stages of the life cycle (Marra et al., 2019)—can provide insights into the interplay of ecological, evolutionary, and anthropogenic influences on migration patterns, habitat use, and coexistence and persistence (Weber & Strauss, 2016; Weber et al., 2017) in a changing Arctic (IPCC, 2014).

Jaegers (skuas outside of North America) are the three smallest *Stercorarius* species, a genus of predatory and kleptoparasitic migratory seabirds that nests in the Arctic (Furness, 1987). Jaegers are the only *Stercorarius* that nest in North America where they play an important regulating role on other taxa within the Arctic tundra summer food web (Gilg et al., 2003; Krebs et al., 2003). Jaegers breed sympatrically across much of the North American Arctic (Furness, 1987) and range in body size: pomarine jaeger (POJA, *S*. *pomarinus*, ~700 g [Wiley & Lee, 2020a]), parasitic jaeger (PAJA, *S*. *parasiticus*, Arctic Skua outside North America, ~450 g [Wiley & Lee, 2020b]), and long‐tailed jaeger (LTJA, *S*. *longicaudus*, ~300 g [Wiley & Lee, 2020c]). Jaeger migrations connect land to sea, and the Arctic region to the tropics (Bemmelen, 2019; Gilg et al., 2013; Troy, 2007). Thus, as congeners that nest sympatrically across most of the Nearctic and then take long‐distance migrations, jaegers could provide a model opportunity for studying Nearctic avian migration.

There are still many existing questions surrounding jaeger marine habitat use. Reviewing many studies of coastal and at‐sea observations, Wiley and Lee (2020b) suggest that during the postbreeding period, PAJA occur more frequently in shallower coastal waters than LTJA (Wiley & Lee, 2020c) or POJA (Wiley & Lee, 2020a), but all species are found in both coastal and open ocean (Furness, 1987). Previous results from light‐level geolocator tracking of jaegers in North America showed that LTJA from the eastern Canadian Arctic over‐wintered off west and southern Africa (Seyer et al., 2021) and PAJA from eastern Greenland migrated to the Caribbean region (Bemmelen, 2019). A presumed‐complete southward migration of a POJA from Alaska, USA, was recorded by satellite and this bird spent the postbreeding period off the coast of southeastern Australia (Troy, 2007). No full‐annual‐cycle migration data have been published for PAJA in North America west of Greenland (Bemmelen, 2019; Wiley & Lee, 2020b) or for any POJA globally (Wiley & Lee, 2020a).

The phylogenetic placement of POJA remains uncertain (Wiley & Lee, 2020a). Multiple lines of evidence suggest that POJA is more closely related to the large skuas that were once classified in a separate genus (*Catharactus*) than to LTJA and PAJA (Andersson, 1999; Chu et al., 2009; Cohen et al., 1997). However, POJA are more similar in size and plumage to the smaller congeners and the three species are sometimes thought of as a guild (Ruffino & Oksanen, 2014). Unlike LTJA and PAJA, POJA do not breed in Greenland or Europe (Furness, 1987; Wiley & Lee, 2020a), creating a break in their breeding distribution in Arctic areas immediately adjacent to the Atlantic Ocean. Pomarine jaegers overwinter on both sides of the Atlantic Ocean (Brown, 1979; Starrett & Dixon, 1947) but the breeding origin of these POJA is unknown. Thus, the break in breeding distribution of POJA relative to LTJA and PAJA leads to questions about whether the three species spread across North America in the same direction(s) from the Palearctic region, their evolutionary origin, and the influences of both biogeography and evolution on contemporary migratory patterns. Broadening the study of jaegers across the North American Arctic could help provide initial answers to these questions.

During the breeding season in North America, pomarine jaegers specialize on cyclic brown lemmings (*Lemmus trimucronatus*) for successful reproduction and thus POJA nesting is generally irruptive—occurring in high density at breeding sites in only some years (Andersson, 1973; Maher, 1974; Pitelka et al., 1955). This observation has implied that POJA are nomadic during the prebreeding period until they find localized areas of high lemming abundance (Wiley & Lee, 2020a), although no direct evidence for nomadic movements of individuals over large areas of the Arctic is known to be available.

We tracked the migrations of sympatrically‐breeding jaeger species from a central Canadian high Arctic nesting location where both Atlantic Ocean and Pacific Ocean destinations seem equally likely. Our goals were to: (1) describe the migratory routes and phenology of movements of the tracked jaegers following sympatric breeding; (2) describe the ocean habitats they used; and (3) provide the first direct information on full‐annual‐cycle movements and nomadism of a pomarine jaeger, an irruptive species whose movements are still largely a mystery (Wiley & Lee, 2020a). Like LTJA tracked from the eastern Canadian Arctic (Seyer et al., 2021), and LTJA and PAJA tracked from Greenland (Bemmelen, 2019), we expected that all three species would spend the nonbreeding period in the Atlantic Ocean. We also expected that PAJA would use shallow, coastal habitats and that LTJA and POJA would use deeper habitats in areas of consistent upwelling. Finally, we hypothesized the POJA would exhibit nomadism in the Canadian Arctic Archipelago prior to nesting and we did not expect the bird to exhibit nest site fidelity.

## 2 METHODS

We captured adult jaegers during incubation (late June to early July) 2018 and 2019 at Nanuit Itillinga (Polar Bear Pass) National Wildlife Area, Bathurst Island, Nunavut, Canada (NINWA, 75°43′N, 98°24′W). Birds were captured with spring‐loaded bownet traps set at nests (*n* = 4), a handheld CO_2_ powered net gun (*n* = 2), or noose mat (*n* = 1). We recorded morphometrics when possible (mass, wing chord, tarsus, bill, and total head plus bill) and fitted birds with a metal band and a color band to aid in identifying individuals.

We used 5 g (LTJA, *n* = 2) and 9.5 g (PAJA, *n* = 2 and POJA, *n* = 1) Argos solar‐powered satellite tags (Microwave Telemetry Inc., deployed 2018–2019) to track seabird movements. Satellite tags were attached using a leg‐loop harness (Mallory & Gilbert, 2008) made of 4.7625‐mm wide tubular Teflon Ribbon (Bally Ribbon Mills) secured with copper crimps. The total tag and attachment weight comprised 0.4%–2.1% of the body mass of known‐weight individuals (Table 1). We assessed wing and leg mobility prior to release and watched birds until they flew out of sight.

**TABLE 1:** Bird morphometrics and details of tracking devices attached to three species of jaegers in the Canadian Arctic Archipelago, 2010–2011 and 2018–2019

Species	Field ID (band number)	Inferred sex	Date deployed (m/d/y)	Bird mass (g)	Culmen (mm)	Total head (mm)	Wing chord (mm)	Diagonal tarsus (mm)	Tag model	Tag mass (g)	Tag % of mass	Tracking duration (days)
Long‐tailed Jaeger	LTJA‐NI‐2019‐01 (1393‐00730)	Female	6/15/2019	U (>300)	27.5	70.6	314	42.9	Argos	5	U	Still transmitting as of 17 November 2021
	LTJA‐NI‐2019‐02 (1393‐00729)	Male	6/23/2019	265	26.3	69.1	300	39.2	Argos	5	1.9%	266
Parasitic Jaeger	PAJA‐NAS‐2010‐01 (794‐63804)	Female	7/12/2010	475	30.9	77.9	344	45.1	GLS	2.1	0.4%	329
	PAJA‐NAS‐2011‐01 (794‐63806)	Male	8/7/2011	510	29	76	330	44.6	GLS	2.1	0.4%	309
	PAJA‐NI‐2018‐01 (894‐51404)	U	6/28/2018	445	29.4	77.8	309	44.3	Argos	9.5	2.1%	303
	PAJA‐NI‐2018‐02 (894‐51401)	Male	7/5/2018	448	28.2	76.9	330	45.8	Argos	9.5	2.1%	236
Pomarine Jaeger	POJA‐NI‐2019‐01 (1015‐00108)	U	7/2/2019	U	37.9	90.7	372	53.7	Argos	9.5	U	361

Note

NI: Nanuit Itillinga (Polar Bear Pass) National Wildlife Area, Bathurst Island, Nunavut, Canada (75°43′17.07″N, 98°24′8.41″W). NAS: Nasaruvaalik Island, Nunavut, Canada (58 km from NI, 75°47′60″N, 96°17′60″W). U: Unknown. Argos: Microwave Telemetry solar‐powered satellite tag. GLS: Lotek light‐level geolocator. For LTJA‐NI‐2019‐01 only the first year of tracking is included in this study.

Data previously collected from two PAJA breeding on nearby Nasaruvaalik Island, Nunavut, Canada (58 km from NINWA, 75°47′N, 96°17′W), were also contributed to this study. These birds were tracked using archival light‐level geolocators (GLS tags) attached with plastic cable ties to darvic leg‐bands (Lotek Inc. LAT2900, 2.1 g). Tags were deployed in July, 2010 (*n* = 1), and June, 2011 (*n* = 1), and recovered the following year by recapturing the birds. Tags also recorded sea surface temperature (SST) when immersed for more than 120 s and stored the minimum daily value.

Area access and animal handling, banding, and tag attachment were approved by Environment and Climate Change Canada (ECCC) Western and Northern Animal Care Committee (Mallory‐EC‐PN‐11‐020, Rausch‐18JR01, Rausch‐19JR01); ECCC Scientific Permits to Capture and Band Migratory Birds (Woodard‐10565N); ECCC Scientific Permits to Kill, Take, Capture, Disturb, or Salvage Migratory Birds/Nests (Mallory‐NUN‐SCI‐09‐01, Rausch‐NUN‐SCI‐17‐03); ECCC National Wildlife Area Permit (Rausch‐NUN‐NWA‐17‐04), and Government of Nunavut Wildlife Research Permits (Mallory‐WL2010‐042, Rausch‐WL2018‐052, Rausch‐WL2019‐041). Animal research was carried out and reported in accordance with relevant guidelines (Fair et al., 2010, Percie du Sert et al., 2020).

### 2.1 Tag programming and processing

Satellite tags were duty‐cycled to maximize solar charging (10 h on, 48 h off). The satellite tag duty cycle resulted in nonregular location estimates during a 10‐h window followed by a maximum gap of 48 h to allow for recharge of the battery via the solar panel. There were occasional gaps in transmissions (“missed duty cycle”). Positions received from Argos were preprocessed with a Kalman filter and delivered with an associated location class indicating the potential error radius and with an estimated error ellipse. Location classes 3, 2, and 1 have an estimated error of 250, 500, and 1500 m, respectively, while the accuracy of auxiliary location classes (0, A, B, and Z) is either variable or unbounded.

Given sampling irregularity and the telemetry error of position estimates, we used a model to estimate most probable paths. We applied the continuous‐time random walk model of Jonsen et al. (2020) using the *foieGras* package in R and estimated movement paths at 24‐h intervals to standardize sampling across birds tracked with different technologies (the maximum resolution of GLS tags was one position per day).

Light‐level data from geolocator tags were initially processed using the manufacturer's built‐in template fit algorithm to estimate locations (the raw light intensities were not stored by this tag model; only the processed position estimates and an estimated error were provided; Ekstrom, 2004). The template algorithm estimates a location once daily by fitting a model for a series of latitudes to light intensities recorded by the tag at a longitude estimated from the time of local noon using the tag's internal clock (Ekstrom, 2004). However, this method was shown to be biased south in winter and north in summer when applied to an Arctic seabird (Frederiksen et al., 2016). We therefore applied a sea surface temperature (SST) correction to further refine position estimates by comparing tag‐collected SST measurements with remotely sensed SST data available for the same dates. We applied an unscented Kalman filter, a state‐space model that incorporates measurement error estimation and the smoothing of the SST field directly in a single model to estimate the most probable track (Lam et al., 2008). We formulated the model with a “solstice” error structure to account for highly erroneous positions near the equinoxes when light level is similar across the globe (defined by the model as September 16–October 2, March 10–March 27) and during which time positions were not estimated. Models were fit using the *ukfsst* package in R.

From the model‐estimated movement paths, we summarized the distance traveled and the duration of time the jaegers spent in breeding, staging, and wintering areas. We calculated the maximum distance reached from nest site, the total distance traveled, and distances of nomadic movements using the *geodist* package in R applied to successive predicted locations using geodesic/great circle distances. For birds tracked with geolocators, positions are unavailable during the breeding season when there is 24‐h daylight, and during equinox periods; reported distances should therefore be considered underestimates. For these birds, we used straight line segments between the nest site and the first estimated location. During the equinox period, we used straight line segments between the estimated locations on either side of the equinox. For satellite tags that ceased transmitting prior to the return northward migration, we added an estimated distance of the return migration to the distance recorded up to the date the tag ceased transmitting. We estimated the expected distance of the return migration if the bird were to retrace its southward migration, omitting stationary periods. For LTJA, we also estimated a return route over the open ocean based on previous studies (Bemmelen et al., 2017; Gilg et al., 2013) and we report the minimum of the two estimates. Given the omission of movements during the staging period, this is most likely conservative. We identified the arrival and departure times to staging and wintering areas based on a combination of net‐squared‐displacement values (birds are resident to an area when the rate of change of net‐squared‐displacement plateaus; Seyer et al., 2021) and visual evaluation of maps.

To describe the jaegers’ marine habitats, we plotted patterns over time of three oceanographic variables commonly used to predict seabird distribution and/or foraging behavior (Tremblay et al., 2009): bathymetry, chlorophyll‐*a*, and sea surface temperature matched to jaeger locations. We used the *rerddapXtracto* package in R to communicate with the NOAA ERDDAP data server (https://coastwatch.pfeg.noaa.gov/erddap) to pull selected oceanographic datasets for the specified location and date. Bathymetry (meters) was derived from ETOPO1, a global relief model with a horizontal grid spacing of 1 arc‐minute (approximately 4 km) developed by the NOAA National Geophysical Data Center. We used chlorophyll‐*a* estimates (chl *a*, mg/m^3^) derived from ocean color data provided by the Visible Infrared Imaging Radiometer Suite (VIIRS) on the Suomi NPP satellite and processed by NOAA. Data were available at a resolution of 4 km. Cloud cover resulted in many missing daily values and we therefore chose to use a weekly composite. For sea surface temperature (SST, °C), we used a blended product from multiple satellite retrievals—the Global Nighttime Foundation Sea Surface Temperature Analysis—produced by the Group for High Resolution Sea Surface Temperature (https://www.ghrsst.org/) and made available by the Jet Propulsion Laboratory Physical Oceanography Distributed Active Archive Center. Unlike other SST datasets, this product provides daily SST estimates for the polar regions and is available on a global 0.054 degree grid. Geolocators collected SST directly, and for these birds, we used tag‐based in situ measurements in lieu of remotely sensed data.

## 3 RESULTS

**FIGURE 1:**
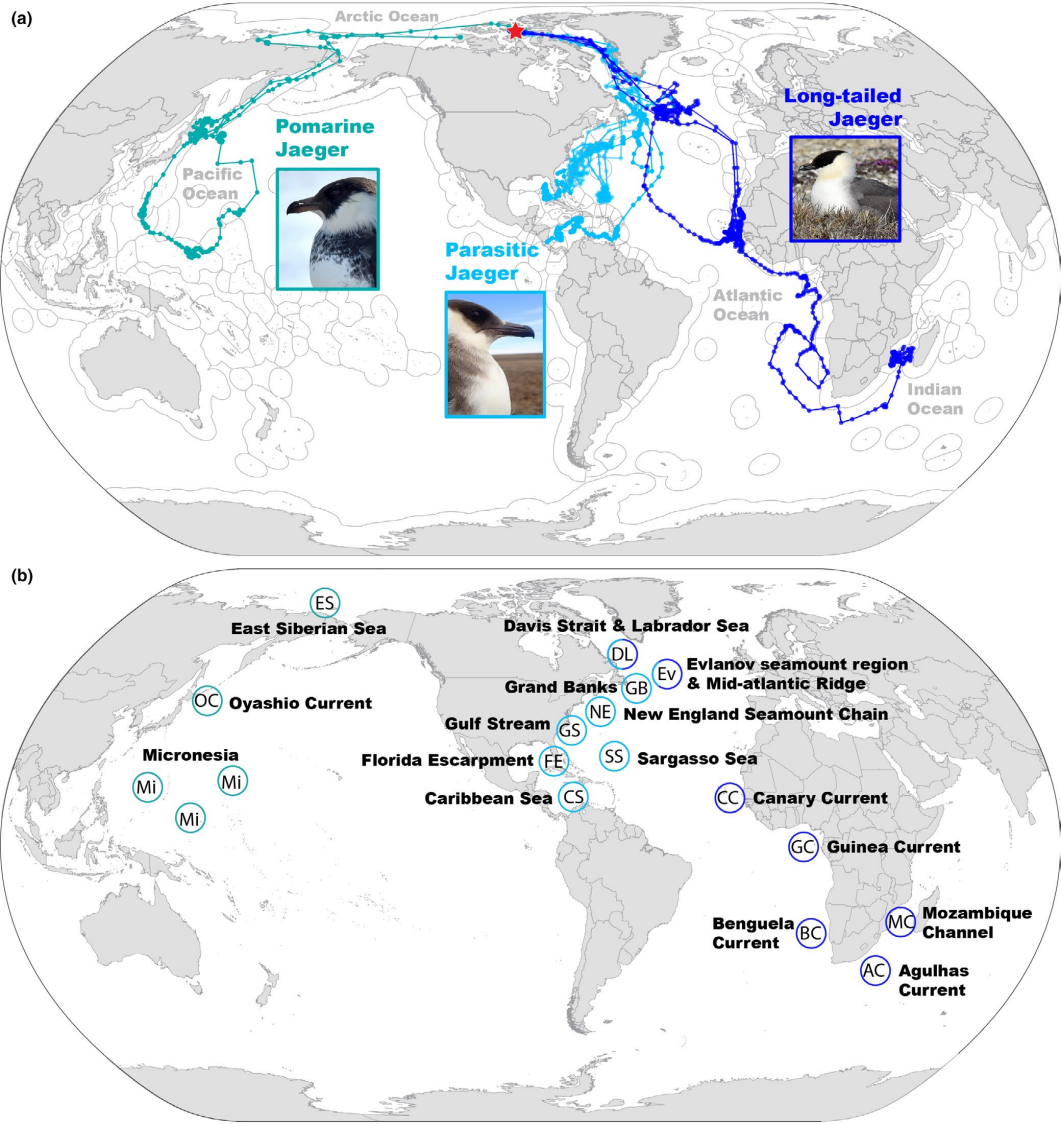
(a) Model‐estimated migration paths of individuals representing three species of jaegers (*Stercorarius* spp.) following sympatric breeding recorded using electronic tracking devices. Red star indicates breeding area in Canada where tags were deployed. Gray outline over the ocean demarcates Exclusive Economic Zones. (b) General locations of staging and wintering areas mentioned in the text color‐coded by the species that used those areas

The three sympatrically nesting jaeger species migrated from central high Arctic Canada to postbreeding habitats in the Atlantic, Arctic, Indian, and Pacific Oceans (Figure 1). Birds departed for migration between July 22 and September 1 (Table 2). Birds were tracked to a maximum distance of 15,418 km straight‐line distance from the nest site (Table 2), and for 236–887 days (Table 1). The tag of one individual remained transmitting as of 17 November 2021 and only the first year of data for this individual is included in this study (LTJA‐NI‐2019‐01).

**TABLE 2:** Estimated distances traveled and annual cycle of seven tracked jaegers from a nest site in Arctic Canada (first year of tracking only)

	Long‐tailed Jaeger (*n* = 2 unless otherwise noted)	Parasitic Jaeger (*n* = 4 unless otherwise noted)	Pomarine Jaeger (*n* = 1)
Distances traveled (km)			
Maximum straight‐line distance from colony	8890–15,418	5705–7146	14,395
Estimated total distance traveled during annual cycle	30,195–57,147^a^	28,648–40,477^a^	41,910
Annual cycle			
Departure from nesting area	22 July	30 Aug.–1 Sept. (*n* = 2)	4 Aug.
Arrival to postbreeding staging area	2–3 Aug.	6–18 Sept. (*n* = 2)	10 Aug./17 Sept.^b^
Departure from postbreeding staging area(s)	20 Aug.–21 Sept.	10 Oct–3 Dec.	1‐Sept./26 Dec.^b^
Arrival to wintering area(s)	5–26 Sept.	19 Oct.−12 Dec	27 Dec.^b^
Departure from wintering area(s)	4 April (*n* = 1)	28 April–7 May (*n* = 2)	6 May
Arrival to prebreeding staging area	22 April (*n* = 1)	13–16 May (*n* = 2)	7 May
Departure from prebreeding staging areas	24 May (*n* = 1)	22–25 May (*n* = 2)	29 May
Breeding season arrival	6–16 June (*n* = 1)^c^	Unknown	June 7–23^d^

Note

For individual metadata, see Table 1. Not all dates during the annual cycle were available for all individuals due to cessation of tag transmissions (LTJA and PAJA) and/or incalculable locations during periods of 24‐h daylight or the equinox periods (Parasitic Jaegers tracked via light‐level geolocators).

^a^ See methods for details of estimates for birds tracked via geolocators and for birds whose tags ceased transmitting during the wintering period.

^b^ The POJA staged for 22 days off Wrangel Island, Russia 10 August–1 September. Sept 17 the bird arrived in the Oyashio Current/Sea of Okhotsk off Hokkaido, Japan where it roamed until December 26 when it commenced a migratory loop over Micronesia. We grouped the movements off of Japan as a staging period because they proceeded a 5‐month migratory loop over Micronesia and the bird also stopped in the Oyashio Current in the spring before returning to the Arctic. However, the movements off Japan could also be considered a first wintering area.

^c^ LTJA‐NI‐2019‐01 arrived at Bathurst Island June 6, 2020, but then immediately departed back to Baffin Bay. The bird returned to Bathurst Island, June 16, 2020.

^d^ June 7 = Date of Arrival to Russia, within known breeding range. June 23 = Date of arrival to Banks Island, Canada, after nomadic movements across terrestrial sites in Russia, June 7–17).

**FIGURE 2:**
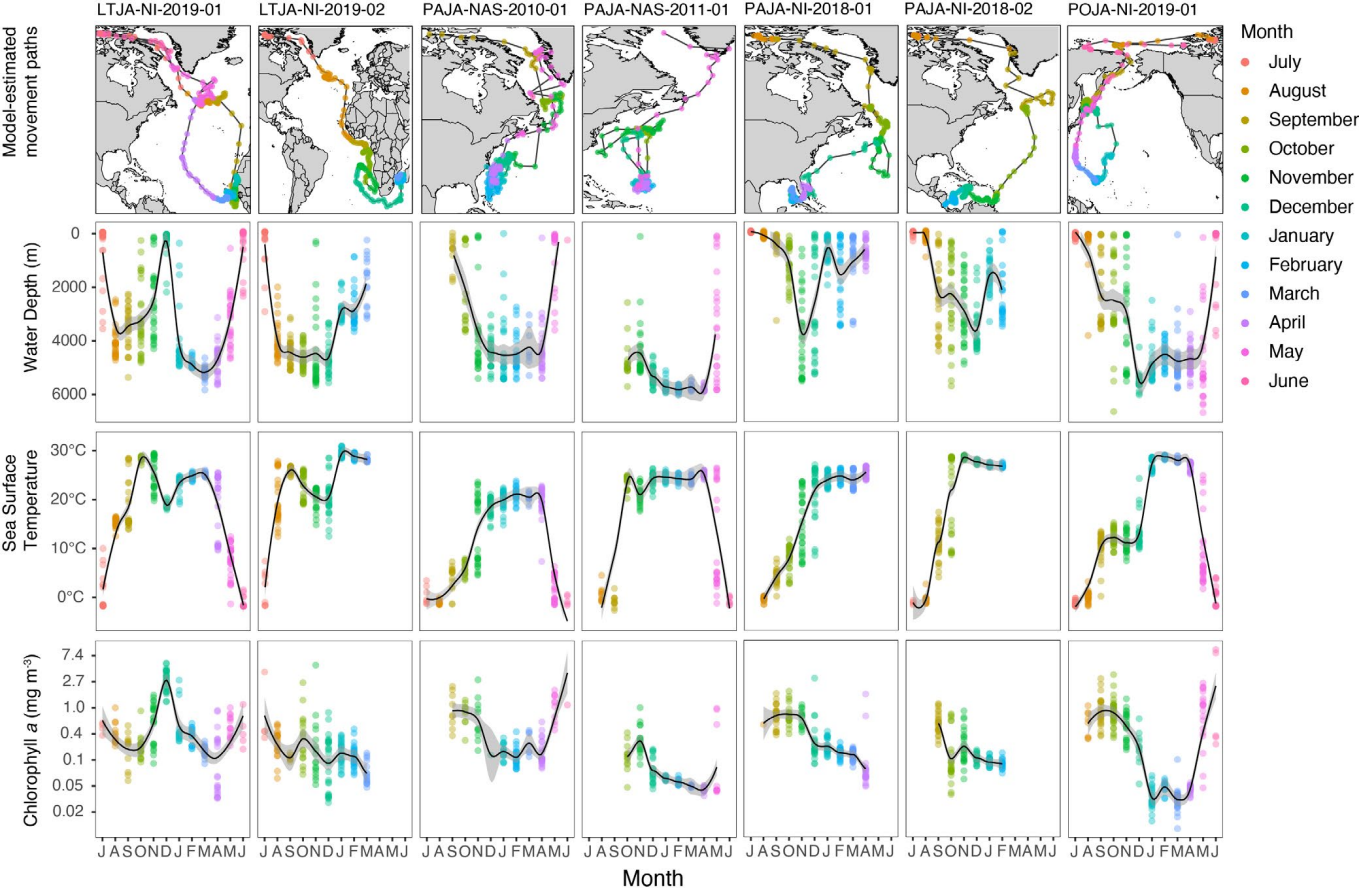
Habitats used by long‐tailed jaeger (LTJA), parasitic jaeger (PAJA) and pomarine jaeger (POJA) electronically tracked following breeding in the Canadian high Arctic. Points on maps indicate model‐estimated daily positions, color‐coded by month. Daily estimates of habitat variables are grouped and colored by month. Bathymetry (water depth), and chlorophyll‐*a* were derived from remotely sensed data (see methods for datasets used). SST was either recorded directly by the tag (GLS tags: PAJA‐NAS‐2010‐01 and PAJA‐NAS‐2011‐01) or were derived from remotely sensed data (Argos satellite tags). Time series begin in July when the birds were incubating eggs and tags were deployed. Solid black lines indicate a loess smooth of the daily estimates and shading around the line indicates the 95% confidence interval

During their migrations, jaegers staged August–December in cool (10–20°C SST), deep marine habitats (2000–4000 m) before overwintering in either tropical (Caribbean Sea, Canary Current, Guinea Current, Micronesian Archipelago), or subtropical (Gulf of Mexico, Guinea Current, Benguela Current, Agulhas Current) marine ecosystems (Figure 2). All LTJA and PAJA migrated to the Atlantic Ocean, staging in some known seabird hotspots (e.g., the North Atlantic Current and Evlanov Seamount [Davies et al., 2021]). However, the POJA migrated west across the Arctic Ocean, where it staged near Wrangel Island, Russia, before continuing to the western Pacific Ocean off Hokkaido, Japan (Figures 1 and 2).

Birds used overwintering habitats September–May (Table 2) including shallow seas, coastal upwelling areas, and oligotrophic and open ocean habitats (Figure 2). LTJA‐NI‐2019‐01 arrived in deep, warm water habitats (23–35°C) off the continental shelf of West Africa in October, shifted to shallow (275 m mean depth) coastal waters of the Canary Current in December corresponding to a peak in chlorophyll (3.11 mg/m^3^), and returned to deep water in January. In contrast, LTJA‐NI‐2019‐02 covered an extensive area in the southeast Atlantic Ocean in September–December including multiple high seas seamount chains as well as the Guinea, Benguela, and Agulhas Currents before moving to warm, shallow water in the Mozambique Channel of the Indian Ocean from January–March when the tag ceased transmitting (Figure 2). The warm wintering habitats used by satellite‐tracked PAJA (22–29°C) generally corresponded with declining chlorophyll throughout the wintering period and use of shallower seas (Figure 2). The POJA overwintered in Micronesia and the western Pacific high seas, where it used deep (4500–5500 m), warm (>25°C), oligotrophic habitats (<0.05 mg/m^3^), and completed a 19,002 km loop migration (Figure 1). The surface chlorophyll values experienced by the bird here were an order of magnitude lower than most habitats used by LTJA and PAJA.

Four individuals were tracked through a full annual cycle (Table 1; Figure 2). LTJA and PAJA each used similar staging areas in postbreeding and prebreeding migrations (Figure 2) but spent less time staging during the prebreeding migration (Table 2). The return route of LTJA‐NI‐2019‐01 was over the central Atlantic Ocean rather than the eastern Atlantic like its postbreeding migration, and the prebreeding route of PAJA‐NAS‐2010‐01 was more coastal than the postbreeding migration (Figure 2).

**FIGURE 3:**
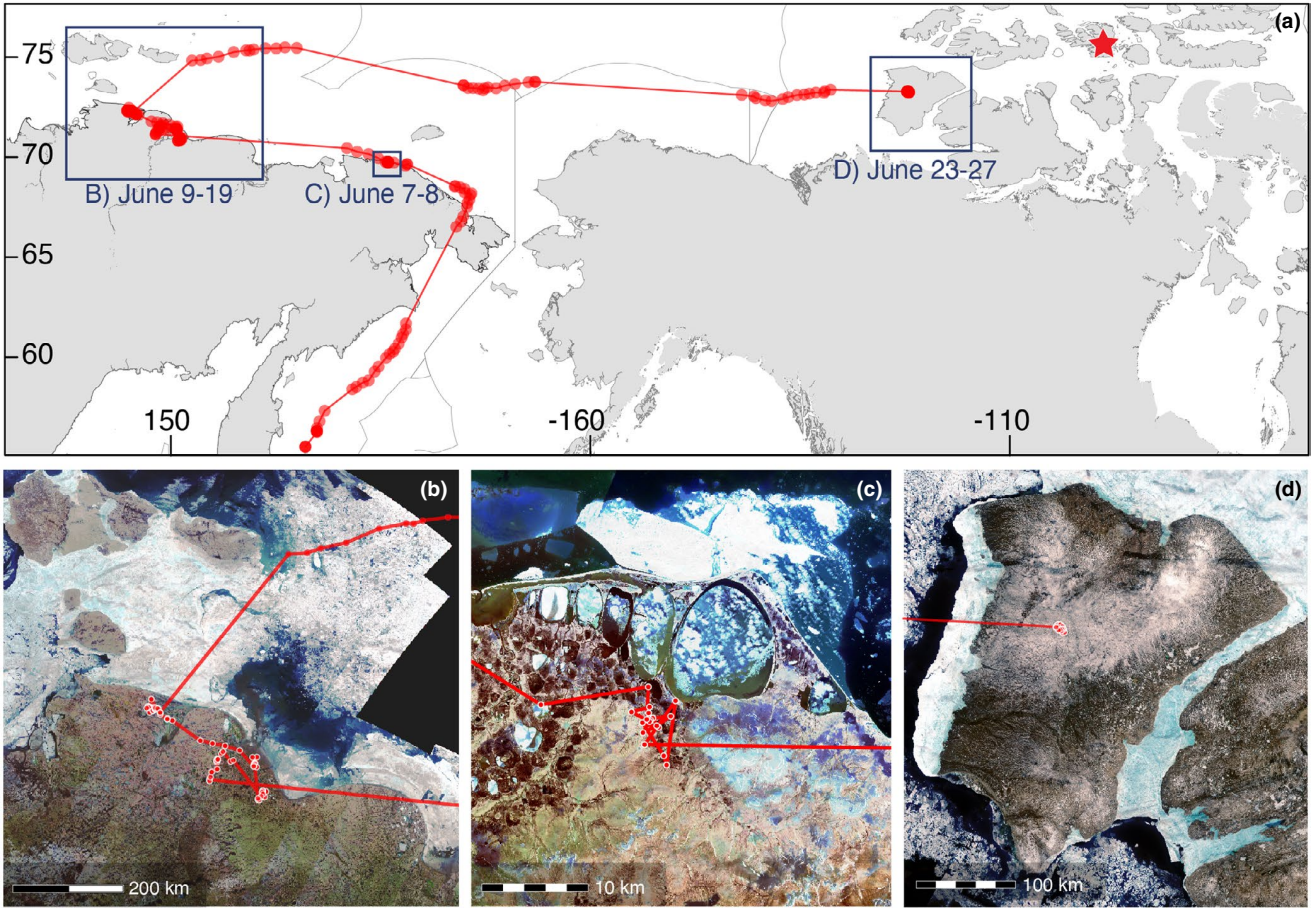
(a) Nomadic movements of pomarine jaeger (POJA‐NI‐2019‐01) tracked via Argos‐satellite tag during a second recorded breeding season (June 1–27, 2020). Red points are model‐estimated locations at the original transmitted timestamps. Red star indicates tag deployment location at 2019 nest site, Bathurst Island, Nunavut, Canada. Gray outline over the ocean demarcates Exclusive Economic Zones. Insets indicate panels b–d. (b) near Billings, Chukotka Autonomous Okrug, Russia (c) near Russkoye Ustye, Sakha Republic, Siberia, Russia. (d) Banks Island, Northwest Territories, Canada (ceased transmission June 27, 2020). Satellite imagery is a composite of images taken by the Copernicus Sentinal‐2 satellite at each location, June 2020

The two PAJA and one LTJA returned to the same nest site in their second recorded year (Figures 1 and 2), whereas the POJA did not return to Bathurst Island in 2020 (Figure 3). After making an overland migration of 253 km, the POJA traveled 5579 km between terrestrial sites in Russia and Canada including an oceanic migration of 3107 km between western Siberia, Russia and Banks Island, Northwest Territories, Canada (121°W, 752 km from 2019 nest site). In Russia, the bird spent 10–12 days visiting multiple sites within the species’ breeding range as far west as 149°E. Russian sites were separated by a linear distance of 1950 km and the total track distance recorded in Russia was 2472 km. The tag ceased transmitting on 27 June, although the onboard activity sensor indicated that tag and bird were still active 23–27 June after arriving on Banks Island, Canada.

## 4 DISCUSSION

We present new findings on the ecology of three species of sympatrically breeding jaegers and further insights into polar migratory divides. From the central Canadian high Arctic, the three jaeger species we tracked visited four oceans and we provide the first direct evidence of extensive terrestrial nomadic behavior of Pomarine Jaeger during the breeding season. While the small sample sizes in our study limit the ability to make statistical comparisons or species‐level inferences, our results provide new information that is a common benefit of animal tracking studies in their initial phase of innovation and discovery (Sequeira et al., 2019).

That the POJA we tracked from the central Canadian Arctic migrated across the Arctic Ocean to the western Pacific Ocean while LTJA and PAJA migrated to the Atlantic Ocean and Indian Ocean is a notable finding. Radar observations at 99°W in the Canadian Arctic suggested that many LTJA and PAJA migrate in a westerly direction (Gudmundsson et al., 2002). The LTJA and PAJA we tracked from a similar longitude (96 and 98°W) migrated east to the Atlantic Ocean and took similar migration routes to LTJA tracked from eastern Canada (81°W and 78°W; Seyer et al., 2021) and LTJA and PAJA tracked from eastern Greenland (24°W and 19°W; Bemmelen, 2019). The POJA in this study migrated west, but large populations of POJA have also been observed on surveys in the Gulf of Mexico (Jodice et al., 2021) and both coasts of the Atlantic Ocean (Brown, 1979; Lee, 1995). The breeding origin of these Atlantic Ocean POJA remains unknown.

From seabird colonies in the central and eastern Canadian Arctic, tracking studies showed that eastern Pacific Ocean destinations were recorded for Thayer's gulls (*Larus thayeri*; *n* = 4 [Gutowsky et al., 2020]), exclusively Atlantic Ocean routes were recorded for Arctic terns (*Sterna paradisaea*; *n* = 22 [Wong et al., 2021]), both eastern Pacific (*n* = 24) and eastern Atlantic destinations (*n* = 2) were recorded for Sabine's gulls (*Larus sabini*) including a mated pair that exhibited a migratory divide (Davis et al., 2016), and from isotopic data, 69% of 167 king eiders (*Somateria spectabilis*) wintered in the North Pacific Ocean while the remainder wintered in the Northwest Atlantic Ocean (Mehl et al., 2004). Our results add new information about jaeger migration routes from a central Canadian site (20 degrees west of previous jaeger tracking studies, [Seyer et al., 2021]) to help further assess the potential for jaeger migratory divides in the Nearctic. Seabird migratory divides to the Pacific and Atlantic Oceans from the high Canadian Arctic Archipelago remain unpredictable.

Our results of disparate migratory routes of sympatrically breeding congeners also raise additional questions about the evolutionary origins (Braun & Brumfield, 1998; Chu et al., 2009; Cohen et al., 1997) and biogeographic spread of North American jaegers. For example, given the observed westerly migration route of the POJA, were POJA populations in central Canada partially populated by individuals originating from westerly populations as glaciers retreated after the last ice age? Do some LTJA and PAJA from the central Canadian Arctic migrate west to the Pacific Ocean as suggested by Gudmundsson's radar observations (Gudmundsson et al., 2002)? Do some POJA observed in the Atlantic Ocean originate from central or western Canada? Given our small sample size, genetic comparisons and additional tracking are needed to answer these questions.

In their geolocator study of the consistency of LTJA migration routes and wintering areas, Bemmelen et al. (2017) suggested that future studies should link location data with oceanography. Here, we provide these first links for a small sample of birds. We found that all birds used both shallow coastal habitats and deep ocean habitats. As in previous studies (Bemmelen, 2019; Bemmelen et al., 2017; Gilg et al., 2013; Simeone et al., 2014; Troy, 2007), jaegers used upwelling habitats of the Benguela Current (LTJA), Canary Current (LTJA), and Oyashio Current (POJA for staging). However, jaegers also used nutrient‐poor habitats. For example, the POJA wandered widely during the postbreeding period over the western North Pacific Sub‐tropical Gyre—an oligotrophic area of low surface chlorophyll, high temperatures, deep water (Karl, 1999), and a place not typically mentioned as a primary overwintering habitat for the species (Furness, 1987; Wiley & Lee, 2020a). The deep water and low productivity habitats used by this POJA also contrasted with the individual Troy (2007) tracked from Alaska that spent its recorded postmigration period in the East Australian Current where dynamic eddies result in localized upwelling and high productivity (Suthers et al., 2011). In addition to describing the oceanographic features jaegers use during the postbreeding period, future studies could evaluate relationships with the distribution of other seabird species that are kleptoparasitized by jaegers and with the distribution of seabird colonies with active nesting during the jaegers’ postbreeding period.

This study is the first to record the full annual cycle of a pomarine jaeger (Wiley & Lee, 2020a). A presumed‐complete southward migration path was reported by Troy (2007) but the bird was not tracked on its return prebreeding migration. Seabirds are known to vary the extent of their movements throughout the breeding season (e.g., incubation vs. late chick‐rearing [Gutowsky et al., 2015]) and long‐distance movements during this period is an expanding area of study for even the smallest birds (Cooper & Marra, 2020). Until this study, only indirect evidence for POJA nomadism existed (reviewed by Wiley & Lee, 2020a) and the geographic extent of nomadism was unknown. During the second recorded breeding season (2020), this individual showed its ability like snowy owls (*Bubo scandiacus*) to prospect across the Arctic presumably in search of lemmings and/or nest sites (Therrien et al., 2014). The international nomadic movements of the POJA in June (5579 km) exceeded the maximum record for the prebreeding nomadic movements of snowy owls (4093 km over 108 days) by 1486 km. Additional tracking is needed to determine if the pattern we observed is representative of the species and to determine the full extent of POJA nomadic behavior.

Distance between Canadian territories in consecutive years was similar to the mean breeding dispersal of 725 km of nine snowy owls (Therrien et al., 2014), although it is unknown whether the POJA in our study initiated a nest in its second year of tracking. In their review of POJA breeding phenology, Wiley and Lee (2020a) noted that most nesting territories in the North American Arctic were established by the third week of June, although some were not established until early July (Bathurst Island peak territory establishment 20–30 June). Maher (1974) observed that transients in Alaska (arriving from at‐sea flocks) also occasionally established short‐term terrestrial territories. Therefore, timing suggests that the POJA in our study arriving at Banks Island, Canada on June 23 could have initiated a nest, but it is equally plausible that these locations represented a transient terrestrial territory of a non‐breeding bird.

Our study modified previous approaches to track jaegers with satellite tags (Seyer et al., 2021; Sittler et al., 2011; Troy, 2007). These modifications may have led to longer tracking durations than were previously attained (maximum 86 days for LTJA and approximately 275 days for POJA), although our small sample size limits general inferences. Rather than a backpack‐style harness that loops over the wings (used previously with LTJA [Seyer et al., 2021; Sittler et al., 2011]), we used a leg‐loop attachment (Mallory & Gilbert, 2008) as had been trialed for POJA (Troy, 2007). For acrobatic birds like jaegers, we felt a leg‐loop harness would have the lowest risk of detrimental impact to the bird but may have a higher risk of being shed (sliding off the tail and legs of the bird possibly due to weight changes or interactions with other birds). For LTJA and POJA, we also used smaller tags than in previous studies: LTJA, 5 g instead of 9.5–10 g (Seyer et al., 2021; Sittler et al., 2011) and POJA, 9.5 g instead of 18 g (Troy, 2007). For LTJA and POJA, our 1% tag and harness to bird mass ratios were conservative in the context of conventional rules for seabird tracking studies (i.e., <3% of the body weight of the bird [Phillips et al., 2003]). To our knowledge, this was the first pilot of satellite tag and harness attachment with PAJA. The 9.5 g tag and harness combination we used for PAJA was <3% of their mass but a 5 g tag would likely have been a better choice due to weight but also due to its lower profile. We have subsequently tracked PAJA from Alaska through full annual cycles (5 of 6 birds) with 5 g tags (1% of body weight, unpublished data, Harrison A‐L. 2021).

Since pelagic seabirds like jaegers spend their postbreeding period at sea, when a tag stops transmitting the reason is often unknown. In this study, the 9.5 g tag included an activity sensor to indicate whether a transmitting tag had stopped moving, but this option was not available on the 5 g tags. Individual PAJA‐NI‐2018‐02 was last tracked at sea during an offshore storm with easterly winds. Final positions were located inland over protected forest in Nicaragua. The tag continued to transmit from this location and the activity sensor indicated it was stationary. Due to the lack of human development in the region and the heavy canopy cover, the solar panel would likely not have charged if the tag was in the forest. Remotely sensed elevation data indicate a constant height of 30–50 m for the month during which the tag was stationary on land. We hypothesize that the tag was dropped solar‐panel facing up, over the trees, or the bird died and was caught by the trees with the tag still attached and facing up.

To evaluate tagging impacts, study designs that include marked but untagged individuals in the same breeding population to allow for comparison of return rates can provide additional understanding. However, for species with low nesting site fidelity like POJA or high natural nest depredation as at Bathurst Island (making capture and resighting difficult), mark–recapture studies may yield few insights.

## 5 CONCLUSIONS

The Arctic region is warming faster than most places on the planet (IPCC, 2014), and even closely related species may respond differently to environmental change (McMahon et al., 2019; Silva et al., 2020; Sun et al., 2017). Transformation of Arctic breeding habitats and disruption to Arctic food webs are thought to be major future threats (Gilg et al., 2012; Ims & Fuglei, 2005). Since lemmings and other small rodents depend for survival on good snow conditions in autumn/winter (Reid et al., 2012), lemming‐reliant species like LTJA and POJA are at special risk of climate change; demonstrated a decline in LTJA in response to collapsing lemming cycles in Greenland. For these three jaeger species breeding in sympatry in the Canadian Arctic, disparate patterns of nomadism and marine migratory connectivity may also have species‐specific management implications (Dunn et al., 2019), although additional tracking is needed to confirm the patterns we observed from a small sample of individuals.

Trends of North American jaeger populations have been largely unknown (Gaston et al., 2009; Wiley & Lee, 2020a, 2020b, 2020c) making it difficult to assess conservation status. Of the three jaeger species, PAJA is of conservation concern in some parts of its range. Globally PAJA is considered stable and is assumed to be the most abundant skua species in the world (Wiley & Lee, 2020b). However, the United Kingdom breeding population has declined steadily since 1986 and more than any other seabird species monitored in the U.K. between 2000 and 2019 (JNCC, 2021). Europe's largest PAJA colony (in Norway) has declined by at least 50% since 1997 (Bemmelen et al., 2021). It is thought that declines are driven by both bottom‐up and top‐down effects including lack of food during the breeding season and nest predation by mammalian and avian predators (Bemmelen et al., 2021; Perkins et al., 2018).

There is good scientific collaboration across the circumpolar Arctic (Davidson et al., 2020). Avian demographic surveys across the North American Arctic region have yielded important insights for shorebirds (Weiser et al., 2020) and have recently met confidence thresholds to provide updated Canadian population estimates for LTJA and PAJA (pers. comm. Smith, P.A. and Rausch, J. 2021). Given that jaegers are critical components of marine and terrestrial food webs that provide ecological connectivity across the world's oceans, we encourage the establishment of a multinational long‐term demographic survey for the jaegers. Finally, our study also contributes to the growing body of literature showing the importance of connectivity across hemispheres and between the coasts and the high seas to migratory seabirds (Beal et al., 2021; Harrison et al., 2018). Demonstrated links between marine biodiversity in the Arctic region and the high seas are timely to inform ongoing negotiations for an internationally binding legal instrument on the conservation and sustainable use of marine biological diversity in the areas beyond national jurisdiction (Popova et al., 2019; United Nations General Assembly, 2017; Vierros et al., 2020).

## CONFLICT OF INTEREST

The authors declare no competing interests.

## AUTHOR CONTRIBUTION

**Autumn‐Lynn Harrison:** Conceptualization (equal); Data curation (lead); Formal analysis (lead); Funding acquisition (equal); Investigation (equal); Methodology (lead); Project administration (equal); Resources (equal); Software (lead); Supervision (equal); Visualization (lead); Writing – original draft (lead); Writing – review & editing (equal). **Paul F. Woodard:** Conceptualization (supporting); Data curation (equal); Investigation (equal); Project administration (equal); Resources (equal); Writing – review & editing (equal). **Mark L. Mallory:** Conceptualization (equal); Data curation (equal); Funding acquisition (equal); Investigation (equal); Methodology (equal); Project administration (equal); Resources (equal); Supervision (equal); Writing – review & editing (equal). **Jennie Rausch:** Conceptualization (equal); Data curation (supporting); Funding acquisition (equal); Project administration (equal); Resources (equal); Supervision (equal); Writing – review & editing (equal).

### OPEN RESEARCH BADGES

This article has earned an Open Data Badge for making publicly available the digitally‐shareable data necessary to reproduce the reported results. The data is available at https://github.com/autumnlynn/SympatricJaegers4Oceans. https://doi.org/10.5061/dryad.nk98sf7v1

## Data Availability

The processed datasets resulting from this study (modeled to account for telemetry error and uncertainty associated with light‐level geolocation, see Methods) are available on Dryad (https://doi.org/10.5061/dryad.nk98sf7v1). The raw datasets contributing to this study are also available publicly under a creative commons license as a part of the Arctic Animal Movement Archive (Davidson et al., 2020) on www.movebank.org (Study Numbers: 973570814, 630339095, 300812056). R code used to conduct the analyses in this study are available on GitHub (https://github.com/autumnlynn/SympatricJaegers4Oceans).
